# Unraveling the nexus between domestic violence and women’s self-rated health status in sub-Saharan Africa: a multi-country investigation for advancing SDG 3 and 5

**DOI:** 10.1186/s12905-025-03935-5

**Published:** 2025-10-27

**Authors:** Alex Bawuah, Michael Sarfo, Jacob Oppong Nkansah, Edward Kwabena Ameyaw

**Affiliations:** 1 School of Global Studies, Faculty of Social Science, University of Sussex, Brighton, UK; 2 School of Human and Health Sciences, University of Huddersfield, Huddersfield, UK; 3 Department of Education Leadership and Policy, The Education University of Hong Kong, Tai Po, Hong Kong SAR, China; 4 Institute of Policy Studies, Lingnan University, Tuen Mun, Hong Kong SAR, China

**Keywords:** Domestic violence, Emotional violence, Physical violence, Sexual violence, Health status

## Abstract

**Background:**

Domestic violence against women (DVAW) is recognised globally as a violation of human rights and a public health concern. This study explores the relationship between DVAW and women’s self-rated health status (SRHS). Identifying how DVAW drives women’s SRHS could direct targeted interventions required to enhance women’s SRHS whilst addressing all forms of DVAW, thereby advancing the course of SDGs 3 and 5.

**Methods:**

The study used data from the Demographic and Health Surveys (DHS) of 5 countries in SSA (Burkina Faso, Côte d’Ivoire, Ghana, Kenya, and Tanzania). 38,882 women (aged between 15 and 49 years) were included in the analysis for the study. SRHS was used as the dependent variable. We focused on four key independent variables: emotional violence, physical violence, sexual violence, and at least one violence. An ordered logistic regression was applied to achieve the study’s objectives.

**Results:**

More than half of the women (53.20%) rated their health status as good. The study highlighted a consistent negative association between various forms of domestic violence and women’s SRHS. Specifically, women who had experienced emotional violence (AOR = 0.76, 95% CI = 0.72–0.80), physical violence (AOR = 0.84, 95% CI = 0.77–0.91), sexual violence (AOR = 0.72, 95% CI = 0.66–0.78) and at least one domestic violence (AOR = 0.77, 95% CI = 0.73–0.80) were less likely to have better SRHS. Furthermore, having high socioeconomic status, consuming fruits and vegetables and not smoking cigarette were positively associated with better SRHS.

**Conclusion:**

Our findings underscore the need for targeted interventions to address the drivers of domestic violence against women. In addition to ongoing awareness campaigns in sub-Saharan Africa, healthcare facilities should implement routine screening for gender-based violence to ensure early support for victims.

## Introduction

Domestic violence (DV) affects all cultures and countries and is a worldwide health priority [1–3]. DV is the intentional and frequently repeated physical, psychological, sexual or economic abuse of a family member by another [4, 5]. “In this study, Domestic Violence Against Women (DVAW) is understood in accordance with the definitions provided by [6, 7] who describe it as intimate partner violence (IPV) by males in formal and informal relationships such as cohabitation, dating, marriage and others. DVAW represents one of the most pervasive and severe types of violence [8–10]. Nearly one in three ever-partnered women and 20% of young women have suffered physical and sexual intimate partner abuse worldwide [7]. The UN Women Gender Data Outlook 2025 highlights that in some Sub-Saharan Africa (SSA) countries, over 40% of women have experienced physical or sexual violence by an intimate partner in their lifetime.

In Sub-Saharan Africa, DV is deeply intertwined with entrenched patriarchal norms that promote male dominance within households and society. The patriarchal system in most SSA societies is reinforced by cultural traditions, religious teachings, and social expectations, all of which contribute to the normalization of male authority and, in some cases, the justification of violence against women [11, 12]. Approximately 24–42% of women in SSA have the notion that wife-beating is sometimes or always justified, with the highest rates observed in countries like Mali (71.1% of women) and lower rates in countries like Malawi (21.4%) [13, 14]. DVAW has implications for meeting the Sustainable Development Goal (SDG) 5.2, which intends to eliminate all forms of violence against women by 2030 [15].

Self-rated health status (SRHS) is a way for individuals to assess their overall well-being, considering physical, mental, social, and functional aspects. It considers personal beliefs, cultural influences, and health-related behaviours [16]. SRHS is a subjective measure that reflects an individual’s internal perceptions and priorities regarding their health. Previous research has shown that people with similar health conditions may evaluate their SRHS differently, indicating that different groups including women victims of DV consider their specific circumstances when evaluating their health [17]. Although healthcare professionals sometimes overlook single-item self-reported scales like SRHS due to contextual factors [18–20], SRHS has proven to be a potent predictor of mortality [21, 22].

Existing research shows widespread adverse health implications and behaviours of DVAW that last a lifetime and generations [9, 10, 22–27]. This can affect how women perceive their health status, as a study by Lancet on systematic analysis for the Global Burden of Disease and another study in Australia have found that DVAW causes more disability-adjusted life years in reproductive-age women [28, 29]. DVAW health-related behaviours associated with SRHS studies often include smoking status, dietary behaviours, physical activity, body mass index (BMI) or presence of obesity, and alcohol consumption [30–32]. Layes et al. determined that individuals who engage in healthy lifestyles are more likely to be pessimistic regarding their health status [33]. Of importance is that there is no straightforward association between SRHS and women victims of DV lifestyle choices. Sociodemographic or cultural variables may influence the relationship between SRHS and behaviour, and seemingly “health-conducive” actions may not yield more positive SRHS [30]. This complexity underscores the need to explore the intricate connection between DVAW and women’s SRHS.

Moreover, although DVAW and its widespread impact remain a pervasive public health issue in SSA, there is limited cross-country comparative research that links DVAW directly to women’s self-assessed health outcomes. Few studies have specifically investigated how experiences of violence influence women’s perceptions of their own health. Self-rated health is a validated indicator of overall well-being and future health outcomes. In SSA, where health data may be fragmented, SRHS serves as a reliable and accessible tool for identifying health disparities and informing targeted interventions, yet its relationship with DVAW remains underexplored in SSA contexts. This study utilizes Demographic and Health Survey (DHS) data from five Sub-Saharan African countries to examine the relationship between domestic violence and women’s self-rated health status, providing a regional and contextual perspectives on this critical public health issue. To expedite the prevention of DVAW and achieve a multi-pronged supportive system, there is a need to understand the association between DVAW and women’s SRHS. Identifying how DVAW drives women’s SRHS would direct targeted interventions required to enhance women’s SRHS whilst addressing all forms of DVAW, thereby advancing the course of SDGs 3 and 5.

## Methodology

### Data source

The study used data from the Demographic and Health Surveys (DHS) of 5 countries in SSA; Burkina Faso (DHS 2021), Côte d’Ivoire (DHS 2021), Ghana (DHS 2022), Kenya (DHS 2022), and Tanzania (DHS 2022). These countries were selected because they are the only countries in sub-Saharan Africa whose DHS data included information on both violence against women and the health status of respondents at the time of the study. In addition to data availability, these countries also provide geographic and sociocultural diversity across West and East Africa, allowing for a broader understanding of how intimate partner violence may affect women’s health status in varying contexts. Furthermore, they represent countries with ongoing efforts to address gender-based violence and improve women’s health, making them relevant case studies for examining the intersection between DV and health outcomes.

The DHS is a nationwide survey that is conducted in over 85 low- and middle-income countries worldwide and follows a consistent protocol and terminology across all countries [34]. It employs a structured questionnaire to gather information on various indicators of health including maternal and child health, fertility, family planning utilization, morbidity, and mortality [34]. The DHS uses a two-stage sampling technique to collect data, starting with the selection of enumeration areas based on each country’s sampling frame, followed by the selection of households from each enumeration area. Detailed information on the sampling and data collection methods can be found in the work of Cameron and Trivedi [35].

The study employed the women’s dataset (IR file) from the DHS. A total of 94,960 women were interviewed (from the pooled sample). Only women who provided responses to questions relating to violence were included in the study. Thus, a final sample size of 38,882 women (aged between 15 and 49 years) was included in the analysis for the study.

### Variables

The dependent variable is the respondent’s self-reported health status. In the survey, the respondents were asked to rate their health status on a 5-point Likert scale with the following responses: 1 = Very bad, 2 = Bad, 3 = Moderate, 4 = Good, and 5 = Very good.

The study used four key independent variables: (i) emotional violence, (ii) physical violence, (iii)sexual violence, and (iv) at least one violence.

Emotional violence was assessed using the DHS variable “d104”, which is coded “1” if the respondent reported experiencing any of the following acts committed by a current or former intimate partner: (i) said or did something to humiliate her in front of others, (ii) threatened to hurt or harm her or someone she cared about, or (iii) Insulted her or made her feel bad about herself. The variable is coded “0” if none of these acts were reported.

Physical violence was assessed using the DHS variable “d107”, which is coded “1” if the respondents reported any of the following severe acts by an intimate partner: (i) kicked, dragged, or beat her up, (ii) tried to choke or burn her on purpose, or (iii) attacked her with a knife, gun, or other weapon. The variable is coded “0” if none of these acts were experienced.

Sexual violence was assessed using the DHS variable “d108”, which is coded “1” if the woman reported any of the following act by an intimate partner: (i) physically forced her into unwanted sex, (ii) physically forced her into other unwanted sexual acts, or (iii) forced her to perform sexual acts she did not want to. A response of “no” to all of these questions results in a code of “0”.

The last variable, “at least one violence” is created from the three variables: emotional violence, physical violence, and sexual violence. This variable was coded “1” if the respondent reported experiencing at least one of the three forms of violence. If the respondent reported no experience of all three forms, the variable was coded 0. This binary indicator captures whether the woman experienced any form of violence, providing a broader measure of exposure.

We further account for following variables: age, education, marital status, employment status, wealth, place of residence, fruits intake, vegetable intake, cigarette smoking, health facility utilisation, and country of residence. The names and definitions of all variables for the study are listed in Table 1.

**Table 1:** Names and definitions of variables used in the study.

Variable Name	Description
Dependent Variable	
Self-rated health status (SRHS)	Respondent rating of their health status 1 = Very bad, 2 = Bad, 3 = Moderate, 4 = Good, 5 = Very good
Independent Variable	
Emotional violence	Respondent experienced emotional violence: 0 = No, 1 = Yes
Physical violence	Respondent experienced physical violence: 0 = No, 1 = Yes
Sexual violence	Respondent experienced sexual violence: 0 = No, 1 = Yes
At least one violence	Respondent experienced at least one violence: 0 = No, 1 = Yes
Control Variables	
Age group	1 = 15–19, 2 = 20–24, 3 = 25–29, 4 = 30–34, 5 = 35–39, 6 = 40–44, 7 = 45–49
Education level	Respondent’s level of education: None = 1, Primary = 2, Secondary = 3, Higher = 4
Marital status	1 = Married, 2 = Living with partner, 3 = Widowed, 4 = Divorced, 5 = Separated
Employment Status	1 = Employed, 2 = Unemployed
Wealth	Household wealth quintile: Lowest = 1, Second = 2, Middle = 3, Fourth = 4, Highest = 5
Residence	Place of residence; 1 = Urban, 2 = Rural
Fruit intake	Respondent taken any fruits in the last 24 h before the survey: 1 = Yes, 2 = No
Vegetable intake	Respondent taken any vegetables in the last 24 h before the survey: 1 = Yes, 2 = No
Smokes Cigarette	Respondent smokes cigarette: 1 = Yes, 2 = No
Health facility use	Has visited a healthcare facility in the last 12 months: 1 = Yes, 2 = No
Country	Country of residence: 1 = Burkina Faso, 2 = Côte d’Ivoire, 3 = Ghana, 4 = Kenya, 5 = Tanzania

### Data analysis

The data were analysed with STATA version 18. Summary tables (percentages) are used to describe the data (Table 2). Given the ordinal nature of the dependent variable (SRHS measured on a 5-point scale from “very bad” to “very good”), the study applied an ordered logistic regression model to evaluate the association between DVAW and women’s SRHS. This model is appropriate for outcomes with natural ordering but unknown spacing between categories. It estimates the likelihood of reporting better (or worse) health status as a function of explanatory variables, while assuming proportional odds across outcome thresholds [36]. The ordered logistic regression model used for the study is specified as follows:

$$\:\text{log}\left(\frac{P\left({Y}_{i}\le\:j\right)}{P\left({Y}_{i}>j\right)}\right)={\alpha\:}_{j}-\:\left({\beta\:}_{1}{DVAW}_{i}+{{\beta\:}_{2}^{\text{T}}X}_{i}\right)\:\text{for}\:j=\text{1,2},\text{3,4}$$

**Table 2:** Descriptive statistics of the sample

	Total Sample	Very Bad	Bad	Moderate	Good	Very Good
Variables	*n* (%)	*n* (%)	*n* (%)	*n* (%)	*n* (%)	*n* (%)
Emotional violence						
No	27,941 (71.9)	39 (0.1)	606 (2.2)	5,376 (19.2)	15,083 (54.0)	6,837 (24.5)
Yes	10,941 (28.1)	36 (0.3)	430 (3.9)	2,753 (25.2)	5,602 (51.2)	2,120 (19.4)
Physical violence						
No	35,083 (90.2)	61 (0.2)	875 (2.5)	7,184 (20.5)	18,713 (53.3)	8,252 (23.5)
Yes	37,97 (9.8)	14 (0.4)	161 (4.2)	945 (24.9)	1,972 (51.9)	705 (18.6)
Sexual violence						
No	36,024 (92.7)	62 (0.2)	904 (2.5)	7,330 (20.3)	19,266 (53.5)	8,463 (23.5)
Yes	2,857 (7.3)	13 (0.5)	132 (4.6)	799 (28.0)	1,419 (49.7)	494 (17.3)
At least one violence						
No	26,669 (68.6)	36 (0.1)	571 (2.1)	5,115 (19.2)	14,374 (53.9)	6,575 (24.7)
Yes	12,213 (31.4)	39 (0.3)	465 (3.8)	3,014 (24.7)	6,311 (51.7)	2,382 (19.5)
Age						
15–19	2,922 (7.5)	21 (0.1)	256 (1.3)	2,498 (13.0)	10,217 (53.0)	6,294 (32.6)
20–24	7,077 (18.2)	26 (0.2)	260 (1.5)	2,820 (16.6)	9,023 (53.1)	4,868 (28.6)
25–29	7,901 (20.3)	28 (0.2)	296 (1.9)	2,769 (18.1)	8,322 (54.3)	3,899 (25.5)
30–34	7,178 (18.5)	30 (0.2)	302 (2.2)	2,730 (20.0)	7,295 (53.4)	3,295 (24.1)
5–39	6,104 (15.7)	27 (0.2)	381 (3.0)	2,914 (23.3)	6,412 (51.2)	2,778 (22.2)
40–44	4,382 (11.3)	25 (0.3)	394 (4.1)	2,513 (26.1)	4,799 (49.8)	1,915 (19.9)
45–49	3,317 (8.5)	28 (0.4)	428 (5.7)	2,362 (31.3)	3,462 (45.8)	1,273 (16.9)
Educational level						
None	13,232 (34.0)	70 (0.3)	967 (3.5)	5,788 (20.8)	14,727 (52.9)	6,276 (22.6)
Primary	11,105 (28.6)	55 (0.2)	721 (2.7)	5,935 (22.2)	14,064 (52.6)	5,946 (22.3)
Secondary	11,198 (28.8)	57 (0.2)	562 (1.7)	5,781 (17.5)	16,840 (50.8)	9,878 (29.8)
Higher	3,347 (8.6)	3 (0.0)	67 (0.9)	1,102 (15.1)	3,899 (53.5)	2,222 (30.5)
Marital status						
Married	23,745 (61.1)	95 (0.2)	1,240 (2.8)	9,266 (20.6)	23,417 (52.2)	10,882 (24.2)
Living with partner	11,397 (29.3)	61 (0.1)	736 (1.8)	7,190 (17.2)	21,905 (52.5)	11,816 (28.3)
Widowed	1,078 (2.8)	9 (0.4)	127 (5.6)	669 (29.7)	1,074 (47.7)	374 (16.6)
Divorced	764 (1.9)	9 (0.5)	60 (3.1)	472 (24.2)	1,022 (52.5)	385 (19.8)
Separated	1,896 (4.9)	11 (0.3)	154 (3.7)	1,009 (24.3)	2,112 (50.9)	865 (20.8)
Employment status						
Unemployed	12,660 (32.8)	74 (0.2)	756 (2.1)	6,141 (17.2)	18,747 (52.4)	10,064 (28.1)
Employed	25,938 (67.2)	111 (0.2)	1,548 (2.6)	12,373 (21.1)	30,439 (52.0)	14,049 (24.0)
Wealth						
Poorest	8,356 (21.5)	51 (0.3)	568 (3.0)	3,894 (20.5)	10,128 (53.3)	4,373 (23.0)
Poorer	7,333 (18.9)	42 (0.2)	545 (3.0)	3,696 (20.6)	9,267 (51.7)	4,383 (24.4)
Middle	7,780 (20.0)	44 (0.2)	482 (2.5)	3,865 (19.9)	10,056 (51.7)	5,021 (25.8)
Richer	8,329 (21.4)	28 (0.1)	416 (2.1)	3,807 (19.2)	10,415 (52.6)	5,122 (25.9)
Richest	7,080 (18.2)	20 (0.1)	306 (1.6)	3,344 (17.8)	9,664 (51.5)	5,423 (28.9)
Residence						
Urban	15,059 (38.7)	70 (0.2)	909 (2.3)	7,157 (18.4)	19,740 (50.8)	10,960 (28.2)
Rural	23,823 (61.3)	115 (0.2)	1,408 (2.5)	11,449 (20.4)	29,790 (53.1)	13,362 (23.8)
Had fruits						
No	26,735 (68.8)	130 (0.2)	1,712 (2.6)	13,400 (20.6)	34,037 (52.3)	15,857 (24.3)
Yes	12,147 (31.2)	55 (0.2)	605 (2.0)	5,206 (17.5)	15,493 (51.9)	8,465 (28.4)
Had vegetables						
No	19,378 (49.9)	87 (0.2)	1,163 (2.5)	9,766 (20.8)	24,707 (52.6)	11,233 (23.9)
Yes	19,486 (50.1)	98 (0.2)	1,153 (2.4)	8,833 (18.4)	24,802 (51.7)	13,077 (27.3)
Smokes cigarettes						
No	28,579 (99.0)	158 (0.2)	1,982 (2.5)	15,896 (20.1)	40,575 (51.4)	20,360 (25.8)
Yes	287 (1.0)	3 (0.4)	28 (3.8)	198 (26.9)	350 (47.6)	156 (21.2)
Used health facility						
No	10,888 (37.7)	74 (0.2)	723 (2.0)	6,538 (18.0)	18,601 (51.2)	10,399 (28.6)
Yes	17,977 (62.3)	87 (0.2)	1,287 (3.0)	9,556 (22.0)	22,324 (51.5)	10,117 (23.3)
Country						
Burkina Faso	9,701 (25.0)	39 (0.2)	642 (3.6)	3,627 (20.5)	9,743 (55.2)	3,608 (20.4)
Côte d’Ivoire	4,514 (11.6)	19 (0.1)	338 (2.3)	2,834 (19.0)	6,823 (45.9)	4,863 (32.7)
Ghana	5,136 (13.2)	73 (0.5)	480 (3.2)	2,648 (17.6)	6,934 (46.2)	4,879 (32.5)
Kenya	15,125 (38.9)	50 (0.2)	710 (2.2)	5,541 (17.2)	17,988 (55.9)	7,867 (24.5)
Tanzania	4,401 (11.3)	4 (0.0)	147 (1.0)	3,956 (25.9)	8,042 (52.7)	3,105 (20.4)

Where $\:{Y}_{i}$ denotes the SRHS (the outcome variable) of individual $\:i$; $\:{\alpha\:}_{j}$ denotes the threshold parameter for the category $\:j$; $\:{DVAW}_{i}$ is a binary variable indicating if individual $\:i$ has experienced domestic violence; $\:{X}_{i}$ is a vector of control variables (age, education, wealth, residence, etc.); $\:{\beta\:}_{1}$ and $\:{\beta\:}_{2}$ are the coefficients to be estimated.

Furthermore, given that DVAW is measured using four variables, we estimated four ordered logistic regression models. The first model examines the association between emotional violence and SRHS. The second model examines the association between physical violence and SRHS. The third model examines the association between sexual violence and SRHS. The fourth model examines the association between experiencing at least one DVAW and SRHS. We adjusted for covariates in all four models. We accounted for the survey design (sample weight, and clustering) in all regression analyses based on the DHS guidelines. Results from all four estimates are provided in Table 3.

**Table 3:** An ordered logistic regression analysis showing the association between DVAW and SRHS of women in sub-Saharan Africa

Variables		Model 1		Model 2		Model 3		Model 4
		SRHS Status		SRHS Status		SRHS Status		SRHS Status
		AOR (95%CI)		AOR (95%CI)		AOR (95%CI)		AOR (95%CI)
Emotional violence								
No								
Yes		0.76 (0.72–0.80)***						
Physical violence								
No								
Yes				0.84 (0.77–0.91)***				
Sexual violence								
No								
Yes						0.72 (0.66–0.79)***		
At least one violence								
No								
Yes								0.76 (0.73–0.80)***
Age								
15–19								
20–24		0.92 (0.83–1.01)*		0.91 (0.83–1.00)*		0.91 (0.82–1.00)**		0.92 (0.83–1.01)*
25–29		0.80 (0.72–0.88)***		0.79 (0.71–0.87)***		0.78 (0.71–0.86)***		0.80 (0.72–0.88)***
30–34		0.72 (0.65–0.80)***		0.71 (0.64–0.78)***		0.70 (0.64–0.78)***		0.72 (0.65–0.79)***
35–39		0.60 (0.54–0.67)***		0.60 (0.54–0.66)***		0.59 (0.53–0.66)***		0.60 (0.54–0.67)***
40–44		0.48 (0.43–0.54)***		0.48 (0.43–0.53)***		0.47 (0.42–0.53)***		0.48 (0.43–0.54)***
45–49		0.37 (0.33–0.41)***		0.36 (0.32–0.41)***		0.36 (0.32–0.41)***		0.37 (0.33–0.41)***
Educational level								
None								
Primary		0.91 (0.85–0.97)***		0.90 (0.85–0.96)***		0.91 (0.85–0.97)***		0.91 (0.85–0.97)***
Secondary		0.96 (0.90–1.03)		0.97 (0.90–1.03)		0.97 (0.91–1.04)		0.96 (0.90–1.03)
Higher		1.05 (0.94–1.19)		1.07 (0.95–1.20)		1.08 (0.96–1.21)		1.05 (0.93–1.18)
Marital status								
Married								
Living with partner		0.80 (0.76–0.85)***		0.81 (0.77–0.85)***		0.81 (0.77–0.85)***		0.81 (0.76–0.85)***
Widowed		0.67 (0.58–0.78)***		0.68 (0.59–0.79)***		0.69 (0.59–0.80)***		0.67 (0.58–0.78)***
Divorced		0.94 (0.80–1.10)		0.90 (0.77–1.06)		0.91 (0.77–1.06)		0.94 (0.80–1.10)
Separated		0.76 (0.67–0.85)***		0.73 (0.65–0.82)***		0.74 (0.65–0.83)***		0.76 (0.68–0.86)***
Employment status								
Unemployed								
Employed		0.97 (0.92–1.02)		0.95 (0.91–1.00)*		0.96 (0.91–1.01)*		0.97 (0.92–1.02)
Wealth								
Poorest								
Poorer		1.11 (1.03–1.19)***		1.10 (1.02–1.18)***		1.11 (1.03–1.19)***		1.10 (1.03–1.18)***
Middle		1.21 (1.13–1.30)***		1.20 (1.12–1.29)***		1.21 (1.12–1.30)***		1.21 (1.13–1.30)***
Richer		1.24 (1.15–1.34)***		1.23 (1.14–1.33)***		1.24 (1.15–1.34)***		1.24 (1.14–1.34)***
Richest		1.36 (1.23–1.49)***		1.35 (1.23–1.48)***		1.35 (1.23–1.49)***		1.35 (1.23–1.49)***
Residence								
Urban								
Rural		1.03 (0.97–1.09)		1.02 (0.96–1.08)		1.02 (0.96–1.08)		1.03 (0.97–1.09)
Had fruits								
No								
Yes		1.20 (1.14–1.27)***		1.19 (1.13–1.26)***		1.19 (1.13–1.26)***		1.20 (1.13–1.26)***
Had vegetables								
No								
Yes		1.05 (1.00–1.10)**		1.05 (1.00–1.09)*		1.05 (1.00–1.10)**		1.05 (1.00–1.10)**
Smokes cigarettes								
No								
Yes		0.76 (0.61–0.94)**		0.76 (0.61–0.95)**		0.77 (0.61–0.96)**		0.76 (0.61–0.95)**
Used health facility								
No								
Yes		0.83 (0.79–0.87)***		0.82 (0.78–0.86)***		0.82 (0.78–0.86)***		0.83 (0.79–0.86)***
Country								
Burkina Faso								
Côte d’Ivoire		1.58 (1.47–1.70)***		1.59 (1.48–1.71)***		1.59 (1.48–1.71)***		1.58 (1.47–1.70)***
Ghana		1.64 (1.52–1.76)***		1.63 (1.51–1.75)***		1.63 (1.52–1.76)***		1.65 (1.53–1.77)***
Kenya		1.24 (1.15–1.34)***		1.25 (1.16–1.35)***		1.24 (1.15–1.34)***		1.26 (1.17–1.36)***
Tanzania		0.87 (0.81–0.94)***		0.89 (0.83–0.96)***		0.90 (0.83–0.97)***		0.88 (0.82–0.95)***
/cut1		0.00 (0.00–0.00)***		0.00 (0.00–0.00)***		0.00 (0.00–0.00)***		0.00 (0.00–0.00)***
/cut2		0.02 (0.02–0.02)***		0.02 (0.02–0.03)***		0.02 (0.02–0.03)***		0.02 (0.02–0.02)***
/cut3		0.24 (0.21–0.27)***		0.24 (0.22–0.28)***		0.24 (0.21–0.27)***		0.24 (0.21–0.27)***
/cut4		2.65 (2.35–3.00)***		2.71 (2.40–3.07)***		2.70 (2.39–3.06)***		2.64 (2.34–2.99)***

*N* 38,882, *AOR* Adjusted Odds Ratio, 95% confidence interval (CI) in parentheses,* Ref *Reference group

*** *p* < 0.01, ** *p* < 0.05, * *p* < 0.10

### Ethical consideration

This study used secondary data obtained from the DHS Program. Access to the data was granted upon request through the DHS Program website (https://dhsprogram.com) after a formal application process. The dataset is anonymised and publicly available to researchers upon approval. As the data are de-identified and collected with informed consent by DHS, no additional ethical approval was required for this analysis. Further details on DHS ethical standards are available at http://goo.gl/ny8T6X.

## Results

### Descriptive statistics

Table 2 presents the socio-demographic characteristics of the study’s sample. More than half of the women (53.20%) rated their health status as good. A higher proportion of the women were aged between 25 and 29 (20.32%). Also, higher proportion of the women had no formal education (34.03%), were married (61.07%), employed (67.20%), lived in rural areas (61.27%) and were in the poorest wealth category (21.49%). Furthermore, 68.76% of the women reported that they had not taken any fruit in the past 24 h before the survey, however, about half of them (50.14%) reported that had taken some vegetables in the past 24 h before the survey. Almost all (99.01%) the women indicated that they don’t smoke cigarette whilst majority had visited a healthcare facility in the last twelve months (62.28%).

**Fig. 1:**
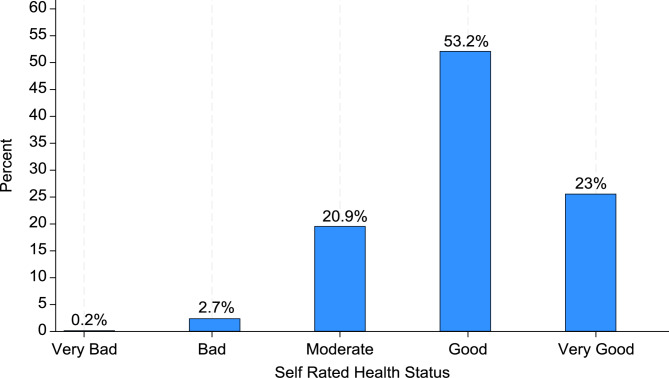
A graph showing SRHS of women from 5 sub-Saharan African countries

28% had experienced emotional violence and 9.77% reported had experienced physical violence. Similarly, 7.35% reported that they were victims of sexual violence. Almost one-third (31.41%) had experienced at least one act of violence. Furthermore, more than half of the women (53.2%) rated their health status as good (Fig. 1).

### Association between DVAW and SRHS of sub-Saharan African women

Table 3 presents the findings of the ordered logistic regression analyses on the correlation between DVAW and SRHS of women from five sub-Saharan African countries (aged 15–49). The result shows that women who experienced emotional violence (adjusted odds ratio [AOR] = 0.76, 95% confidence interval [CI] = 0.72–0.80) were less likely to rate their health status positively (from very bad to very good). Similarly, women who experienced physical violence were less likely to have better SRHS (AOR = 0.84, 95% CI = 0.77–0.91). Likewise, those who experienced sexual violence were less likely to have better SRHS (AOR = 0.72, 95% CI = 0.66–0.79). Also, women who experienced at least one episode of domestic violence were less likely to have better SRHS (AOR = 0.77, 95% CI = 0.73–0.80).

Age, marital status, wealth status, fruit intake, vegetable intake, smoking status and health facility visit emerged as significant predictors of SRHS across all four models. However, we report the results from model 4 as that provides an aggregated findings from all the domestic violence indicators. The result (from model 4) shows that the odds of having a better SRHS decrease as age increases and this was phenomenal among those aged 45–49 (OR = 0.37, 95% CI = 0.33–0.41) were less likely to have better health status than those aged between 15 and 19.

The results further revealed that. compared to the married women, women who are not currently married are less likely to report better SRHS, particularly widowed women (AOR = 0.67, 95% CI = 0.58–0.78). Relative to poorest women, the odds of having a better SRHS were higher for women in all wealth categories, particularly for the richest women (AOR = 1.35, 95% CI = 1.23–1.49). Women who consumed fruits in the past 24 h prior to the survey were more likely to have better SRHS (AOR = 1.20, 95% CI = 1.13–1.26). Similarly, women who had taken vegetables in the past 24 h prior to the survey were more likely to have better SRHS (AOR = 1.05, 95% CI = 1.00–1.10). Cigarette smokers were less likely to have better SRHS (AOR = 0.76, 95% CI = 0.61–0.95). Also, those who had visited a healthcare facility in the last twelve months were less likely to have better SRHS (AOR = 0.83, 95% CI = 0.79–0.86).

## Discussion

The objective of this study was to assess the association between DVAW and women’s SRHS in SSA. In this section, we summarise key patterns, offer plausible explanations for observed associations, compare our findings with other existing studies, and acknowledge the study’s limitations. Consistently, our study found that all four primary forms of violence; emotional, physical, sexual and at least one type of violence were statistically significant predictors of poorer SRHS. Additionally, age, marital status, wealth status, fruit and vegetable consumption, cigarette smoking, and recent health facility use were also noted as significant correlates of SRHS.

Consistent with our findings, which indicate that more than half of the women (53.20%) assessed their health as very good, a study by McKelvie et al. examining the health outcomes of women with a history of domestic violence in Sanma Province reported that 50% of the respondents rated their health outcomes as excellent [36]. A possible explanation for the consistent negative association between DVAW and women’s SRHS lies in the psychosocial and physiological effect of abuse. Violence, especially emotional and sexual, is known to cause sustained psychological distress, low self-worth, social withdrawal, and heightened stress responses. Consequently, women’s subjective evaluations of their health may reflect cumulative trauma, even in the absence of acute physical symptoms.

The study highlighted a consistent negative association between various forms of DV - namely emotional, physical, and sexual violence - and women’s SRHS status. Specifically, emotional violence, physical violence, sexual violence, and a history of domestic violence were all significantly associated with poorer SRHS of the women. These findings are consistent with previous studies which have documented similar associations between DV and women’s health [37–41].

Of particular concern is the finding about emotional violence, which encompasses verbal abuse, humiliation, rejection, and demeaning behaviours. Despite being often overlooked, emotional violence poses a significant threat to women’s health, especially their mental well-being. This is particularly relevant in traditional African societies, where women may internalise feelings of worthlessness and hopelessness due to societal norms and expectations [42, 43].

Similarly, women who had experienced sexual violence within the context of DV had an increased risk of reporting poor SRHS. This aligns with findings from the World Health Organization (WHO) [43], as well as studies by Bonomi et al. [44] and Thompson et al. [45]on violence against women. This finding may be attributed to factors such as lack of family support, feelings of guilt and shame from sexual abuse, and fear of societal judgment, which inhibit victims from seeking necessary care and support. Additionally, in Africa, cultural norms restricting discussions on sexual encounters outside marital relationships exacerbate these challenges [42]. This cyclical psychological trauma can overshadow well-being and lead to poor self-ratings of health status.

It is widely acknowledged that low socioeconomic status is closely linked to a higher incidence of DV [46]. Notably, we found that women from more affluent backgrounds exhibited greater odds of rating their health status relatively better compared to their less privileged counterparts. This disparity can be attributed to the greater resources available to wealthier individuals, enabling them to access essential healthcare services, counselling, and health-enhancing products following experiences of domestic abuse [47–49]. In contrast, women from disadvantaged backgrounds may face barriers to accessing comprehensive healthcare, exacerbating the health implications of their victimisation [50].

Furthermore, our study highlights the positive impact of dietary habits on SRHS. Specifically, we found that individuals who consumed fruits and vegetables had higher odds of rating their health as very good. This finding is consistent with previous studies, which have demonstrated a positive association between fruit and vegetable consumption and women’s health [51]. The health benefits of fruits and vegetables, such as their immune-boosting properties, regulation of healthy blood pressure, and antioxidant effects, may contribute to improved health outcomes among victimised women. Notably, these benefits are particularly pertinent in mitigating the negative health impacts of stress hormones induced by domestic abuse [52].

Our research findings illuminate a notable association between domestic violence and the self-rated health status (SRHS) of unmarried women. Our analysis revealed that unmarried women were less likely to report a positive self-rated health status compared to their married counterparts. This finding underscores the role of marital status in shaping women’s health perceptions, especially in the context of domestic violence. A plausible explanation for this is that unmarried women may experience reduced social support, greater economic vulnerability, and limited access to consistent healthcare, which can exacerbate both physical and psychological stressors and negatively influence their perception of health. This is consistent with Lamidi [53], who reported that never-married adults experienced declining trends in self-rated health between 2000 and 2018 compared to those in marital unions.

We noted an inverse relationship between age and SRHS, whereby women aged 45–49 were associated with poorer SRHS. The existing literature supports the notion that as women age, they experience a complex interplay of psychological, cognitive, and social changes, which can significantly shape their subjective perceptions of health [54, 55]. For example, the social support theory, particularly the buffering support model, suggests that individuals with high levels of perceived social support tend to experience fewer negative effects following stressful events, such as domestic abuse, compared to those lacking support [56]. This is particularly relevant in the African context of aging, where women often find themselves overwhelmed by family responsibilities and facing increasing isolation [57]. Consequently, loneliness becomes more prevalent, and the combined impact of domestic abuse, loneliness, and the heightened risk of depression and anxiety, exacerbated by a lack of social bonding capital in older age, may contribute to a nuanced of how perception of health status evolves over the aging process [58, 59].

Additionally, our analysis revealed that smoking was associated with a lower likelihood of reporting better SRHS among victimised women. Smoking has been well-documented to have detrimental effects on overall health, including increased risk of chronic diseases, impaired immune function, and accelerated aging processes [60, 61]. Therefore, the observed association between smoking and poorer SRHS underscores the importance of addressing modifiable health behaviours in interventions aimed at improving the health outcomes of domestically victimised women.

### Strengths and limitations

Our study utilized a large sample size from five Sub-Saharan African countries, enhancing the power of detecting associations. However, this study is not without limitations, such as the use of cross-sectional data and reliance on self-rated health status, which is more subjective. Also, this is a cross-sectional study, hence causal inference cannot be drawn from our findings. Additionally, one limitation is that pooling data from five countries may obscure national differences, creating a false impression of homogeneity among women in sub-Saharan Africa, which may not reflect the actual diversity of experiences.

## Conclusion

In conclusion, our study has highlighted the association between DV and SRHS of victimised women in five SSA countries. Our study found that emotional, physical, sexual, and any form of violence were significant predictors of poorer self-rated health (SRH). Additionally, age, marital status, wealth, fruit and vegetable intake, smoking, and recent use of health facilities were also significantly associated with SRH.

Our study’s findings have underscored the pervasive negative associations between DVAW and women’s perception about their health status. These findings support the urgent need for holistic strategies that involve healthcare providers, legal systems, mental health professionals, community organisations and policymakers to address DVAW and its detrimental effects on women’s health by focusing on key interventions such as psychosocial counselling, community -based support, nutritional education, legal protection and law enforcement and public health campaigns. Efforts should focus on raising awareness about the various forms of violence against women, providing accessible support services for victims, and implementing policies to prevent and respond to gender-based violence effectively.

## Data Availability

The dataset used for this study is available in a public, open access repository. The data set can be accessed via https://dhsprogram.com/data/available-datasets.cfm.

## Abbreviations

AIDS: Acquired Immune Deficiency Syndrome
AOR: Adjusted Odds Ratio
BPV: Bleeding Per Vagina
CI: Confidence Interval
DHS: Demographic Health Survey
DV: Domestic Violence
DVAW: Domestic Violence Against Women
HIV: Acquired Immunodeficiency Virus
IPV: Intimate Partner Violence
PSTD: Post Traumatic Stress Disorder
SDG: Sustainable Development Goals
SRHS: Self-Rated Health Status
WHO: World Health Organisation
